# Draft Genome Sequence of *Enterococcus faecalis* Strain F165 Isolated from a Urinary Tract Infection

**DOI:** 10.1128/genomeA.01084-16

**Published:** 2016-10-06

**Authors:** Thiago G. S. Paim, Luiza Pieta, Janira Prichula, Gustavo E. Sambrano, Renata Soares, Aline Dall Bello, Jeverson Frazzon, Pedro A. d’Azevedo

**Affiliations:** a Laboratory of Molecular Microbiology, Federal University of Health Sciences of Porto Alegre, Porto Alegre, Brazil; b Institute of Food Sciences and Technology, Federal University of Rio Grande do Sul, Porto Alegre, Brazil

## Abstract

We report here a draft genome sequence of *Enterococcus faecalis* strain F165 isolated from a urine specimen in South Brazil. The genome size was 3,049,734 bp, with a G+C content of 37.38%, and genes related to antimicrobial resistance and adherence were found in the strain. These findings are consistent with pathogenesis of *E. faecalis* species.

## GENOME ANNOUNCEMENT

*Enterococcus faecalis* strains are the Gram-positive cocci often recovered from urinary tract infections (UTIs), particularly among individuals that have risk factors such as advanced age, pregnancy, or urinary catheterization. These Gram-positive cocci are isolated from polymicrobial communities on the surface of indwelling urinary devices, causing at least 30% of catheter-associated UTIs (1). Although enterococci are commensals in the gastrointestinal tract*, E. faecalis* is known as an opportunistic pathogen causing infections related to health care, mainly due to the expression of virulence factors associated with adherence of mucosal and abiotic surfaces (2).

We report here a draft genome sequence of a clinical isolate of vancomycin-susceptible *Enterococcus faecalis*, namely, F165, recovered from positive uroculture in a tertiary care hospital of South Brazil.

Genomic DNA was extracted using the Wizard genomic DNA purification kit (Promega). The quality and yield were assessed by agarose gel electrophoresis and QuBit double-stranded DNA (dsDNA) high-sensitivity (HS) assay kit (Life Technologies), respectively. The library for sequencing was prepared using the Nextera XT DNA kit and index primers (Illumina), and the reads were generated by MiSeq reagent kit version 2 with 300 cycles on an Illumina MiSeq platform. A total of 757,793 paired-end reads were recovered, and the sequences were adapter and quality trimmed using FASTX-Toolkit (http://hannonlab.cshl.edu/fastx_toolkit/index.html). All the reads showed quality score *Q* > 30. A shell script command for Linux/Unix was carried out to *de novo* assembly using the following assemblers: ABySS (3), SPAdes (4), SOAP*denovo*2 (5), and Velvet (6), with k-mer sequence length ranging from 20 to 63. The best assembly had a 37-mer and was performed on ABySS. Then, the NCBI Prokaryotic Genome Annotation Pipeline (7) was used to annotate the DNA sequences. The draft genome has 3,049,734 bp in a total of 194 contigs, with an *N*_50_ of 37,159 bp and G+C content of 37.38%. The annotation found 2,886 coding sequences, 29 tRNAs, and six rRNAs.

The identification of acquired antibiotic resistance and virulence factor genes were performed with the Web tool ResFinder and VirulenceFinder, respectively (8, 9). Four genes to aminoglycoside resistance were identified [*ant(6)-Ia, aac(6′)-aph(2′′), aph(3′)-III,* and *str*], which was in agreement with the phenotype screening for high-level aminoglycoside resistance. Ciprofloxacin and norfloxacin resistance have been found in the strain, and mutations that confer amino acid substitutions in housekeeping genes, such as DNA gyrase A (*gyrA*), are the main mechanism of antimicrobial nonsusceptibility to fluoroquinolone agents (10). Therefore, pairwise alignment was performed with Clustal W algorithm between *gyrA*-translated genes of *Enterococcus faecalis* ATCC 29212 and *E. faecalis* F165 strains. The serine at position 84 (relative to amino acid coordinates of GyrA from F165 strain) was changed to isoleucine, similar to a previous study in resistant strains (11). Fifteen virulence factors were found, such as aggregation substance (*agg*), collagen adhesin (*ace*), hyaluronidase (*hylA* and *hylB*), endocarditis- and biofilm-associated pili (*ebpA*, *ebpB*, and *ebpC*), and *E. faecalis* endocarditis antigen A (*efaA*fs) (2). All these genes contribute to adherence, aggregation, and invasion of the host tissue for enterococcal strains.

### Accession number(s).

This Whole Genome Shotgun project has been deposited at DDBJ/ENA/GenBank under the accession MBRC00000000. The version described in this paper is version MBRC01000000.
